# Effectiveness Evaluation of Silicone Oil Emulsion In Situ Polymerization for Dehydration of Waterlogged Wooden Artifacts

**DOI:** 10.3390/molecules29204971

**Published:** 2024-10-21

**Authors:** Mengruo Wu, Xiangna Han, Zhiguo Zhang, Jiajun Wang

**Affiliations:** 1 Key Laboratory of Archaeomaterials and Conservation, Ministry of Education, Institute for Cultural Heritage and History of Science & Technology, University of Science and Technology Beijing, Beijing 100083, China; wumengruo@163.com; 2 National Center of Archaeology, Beijing 100020, China; wangjiajun@caf.ac.cn

**Keywords:** silicone oil, emulsion, in situ polymerization, waterlogged wooden artifacts, dehydration, conservation materials

## Abstract

Organosilicon materials have shown potential as dehydration agents for waterlogged wooden artifacts. These materials can polymerize under normal conditions to form polymers with favorable mechanical strength, antibacterial properties, and aging resistance. However, the insolubility of most organosilicon hindered their penetration into waterlogged wood, which may lead to an unwanted cracking. This study aimed to evaluate the effectiveness of polydimethylsiloxane (PDMS) and hydroxy-terminated polydimethylsiloxane (PDMS-OH) with low viscosity and moderate reactivity for dehydrating waterlogged wooden artifacts from the *Nanhai No.1* shipwreck. Four surfactants ((3–aminopropyl) triethoxysilane (APTES), alkyl polyoxyethylene ether (APEO), tri-methylstearylammonium chloride (STAC), and fatty alcohol polyoxyethylene ether (AEO)) and cosurfactant were employed to transform the two kinds of water-repellent silicone oils into eight groups of highly permeable oil-in-water (O/W) emulsions. Under the catalysis of a neutral catalyst, in situ polymerization occurred within the wood cells. Group P2-2 formulated with PDMS-OH and APEO showed the best efficiency in maintaining the dimensions of the wood during dehydration. The dehydrated wood exhibited a natural color and texture with a minimal volume shrinkage rate of 1.89%. The resulting polymer adhered uniformly to the cell walls, effectively reinforcing the wood cell structure. The weight percent gain of the wood was only 218%, and the pores of the cell lumen were well maintained for future retreatment. This method effectively controlled the sol–gel reaction process of the organosilicon and prevented damage to the wooden artifact during the dehydration process. Moreover, the dehydrated wood samples only experienced a low weight gain of 17% at 95% relative humidity (RH), indicating their great environmental stability.

## 1. Introduction

Archaeological wooden artifacts are rich in historical information and provide invaluable evidence for the study of the technological, artistic, and cultural aspects of ancient human societies [1,2]. However, due to degradation from combined chemical and biological factors, waterlogged archaeological wood usually exhibits high decay levels and complex degradation patterns [3]. The structural integrity of the wood cell walls made the physical and mechanical properties of the wood severely compromised [4,5,6]. The breaking of hydrogen bonds within the polysaccharide framework and cellulose network leads to the degradation of cellulose and hemicellulose [7]. Additionally, the substantial degradation of hemicellulose acetyl side chains, β–O–4 linkages, and the oxidation and demethylation of lignin also contribute to the deterioration process. All these degradations resulted in the loss of the structural integrity of wood cells, creating numerous pores occupied by free water within the wood [8,9,10]. Consequently, waterlogged archaeological wood typically exhibits increased porosity and a loose, sponge-like structure, which is easy to break down once it has gone through the dehydration process [11].

Therefore, dehydration is a critical step in the conservation of waterlogged wooden artifacts [12]. Researchers continuously seek dehydration materials that offer superior dimensional stability, high permeability, efficient dehydration, low hygroscopicity, and enhanced durability [13,14]. Organosilicon materials, known for their high strength, water resistance, antibacterial properties, and aging resistance [15], have found applications in wood modification and waterproofing [16]. These materials are also highly modifiable [17], with reports of silicone oil being used to prepare wood-based biomimetic materials [18,19]. In recent years, they have also been found to hold significant potential in cultural heritage conservation [20,21].

The application of organosilicon materials to waterlogged wooden artifacts involves in situ polymerization to support and reinforce the wood matrix. Currently, the reported organosilicon materials for dehydrating archaeological wood primarily include siloxane monomers, silane coupling agents, and silicone oils. Studies have shown that in waterlogged wood, organosilicon materials exhibit efficient sol–gel transition [22,23]. This property is advantageous for the dehydration and reinforcement of severely degraded waterlogged archaeological wood but also means the polymerization is more difficult to control.

Among the earliest attempts was the use of tetraethoxysilane (TEOS), which was commonly employed in stone artifact conservation. Although TEOS monomers provided good dimensional stability and reinforcement for wood, the stress generated during the sol–gel process could lead to wood cracking [24]. Consequently, organic segments need to be introduced to enhance the toughness of the gel network structure. In the early 1990s, researchers used ethanol solutions of methyltrimethoxysilane (MTMOS/MTMS) and 2–(heptadecafluorooctyl) ethyltrimethoxysilane (HFOETMOS) to impregnate wood under vacuum conditions, achieving improved waterproofing, dimensional stability, and UV resistance [25]. Additionally, resistance to decay fungi was also enhanced [26,27]. Recently, Broda et al. evaluated the dehydration and reinforcement effects of a dozen organosiloxanes containing various functional groups on waterlogged wooden artifacts. Among these, 3-mercaptopropyltrimethoxysilane (MPTES) demonstrated the best results for dimensional stability [28,29]. Hamilton’s conservation manual introduces a method for protecting archaeological wood using a mixture of silicone oil and isobutyltrimethoxysilane. This method also requires ethanol and acetone to remove water from the wood, and due to the weaker reactivity of silicone oil compared to silane, the polymerization of silicone oil is catalyzed at 52 °C [14].

Existing studies indicate that the volumetric shrinkage rate of waterlogged archaeological wood treated with organosilicon materials can be reduced to approximately 2% to 5%. Post treatment, organosilicon primarily undergoes polymerization and solidification within the cell walls of the wood, preserving the natural porous structure of the cell cavities [30,31,32]. However, these methods require substantial use of organic solvents, posing significant limitations for practical applications. To eliminate the need for organic solvent replacement, the insolubility of organosilicon in water presents challenges in penetration and rapid surface polymerization. An effective way to solve this problem is to prepare aqueous dispersions or emulsions from insoluble substances [33,34].

In this study, two low-molecular-weight silicone oils were selected as the primary agents. Four commonly used surfactants were utilized to encapsulate the organosilicon within microspheres formed by the surfactants through an emulsification process, creating a water-in-oil (O/W)-type silicone oil emulsion that is easily soluble in water. This approach aimed to achieve the direct penetration and water displacement of organosilicon in waterlogged wooden artifacts. Subsequently, with the catalysis of dibutyltin dilaurate (DBTL), in situ polymerization of the silicon oil on the wood cell walls was expected to occur, to achieve dehydration and stabilization by supporting the cell wall structure and sealing the hydroxyl groups.

## 2. Results

### 2.1. Silicone Oil Emulsions

**Figure 1:**
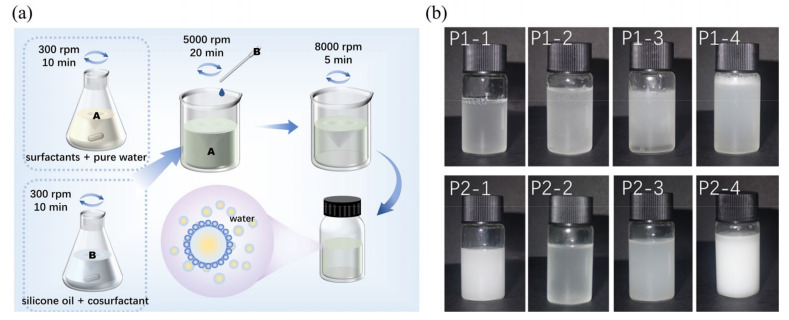
(**a**) Schematic diagram of the silicone oil emulsion preparation process. (**b**) The 8 groups of silicone oil emulsions.

The PDMS emulsions were marked as group P1, and the PDMS-OH emulsions were marked as P2. Four different commonly used surfactants were introduced to the two kinds of silicon oils (Figure 1a) and prepared into eight groups of emulsions numbered as P1-1 to P1-4 and P2-1 to P2-4, respectively. The resulting emulsions exhibited a milky white or semi-transparent appearance, as illustrated in Figure 1b. We found that the viscosity of the emulsions was predominantly influenced by the kind of surfactant we used but not the silicone oil (Table 1). The viscosity of the emulsion was minimal in the case of the STAC (P1-4 and P2-4), imparting a high degree of flowability. However, emulsions formulated with AEO (P1-3 and P2-3) exhibited significantly high viscosity up to 105 mPa·s, which even resembled a gel-like consistency. The viscosity of the emulsions in the remaining groups fell within a moderate range, closely approximating the viscosity of pure silicon oils (~40 mPa·s). The overall variation was minor while PDMS emulsions had slightly higher viscosity than HPDMS-OH emulsions due to their molecular weight differences.

### 2.2. Physical Properties and Appearance

**Figure 2:**
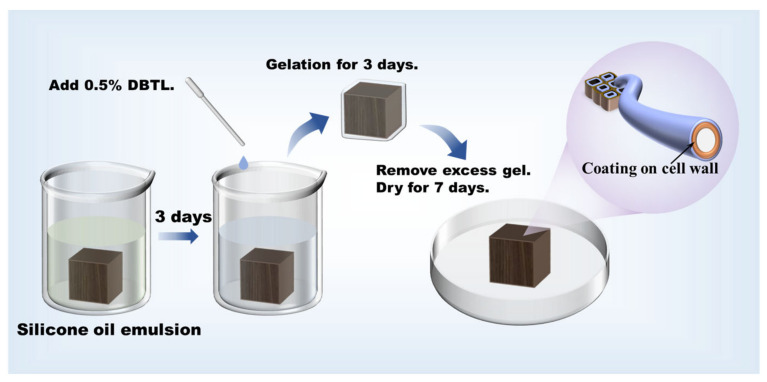
Schematic diagram of the dehydration process of waterlogged archaeological wood.

The waterlogged wood was cut into sample cubes of 1 cm^3^ and separately immersed in the eight groups of silicone oil emulsions for 3 days. Then, dibutyltin dilaurate (DBTL) was added to the emulsions for catalytic polymerization. After 3 days of reaction, the wood samples were cleaned and thoroughly dried together with the untreated sample in a normal condition. The dehydration process was as shown in Figure 2.

**Figure 3:**
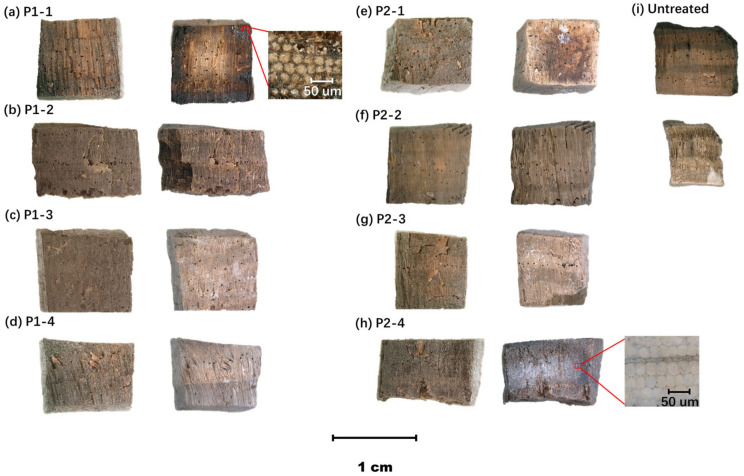
The dehydrated pine wood samples before (**left**) and after (**right**) treatment with silicone oil emulsions. (**a**) P1-1; (**b**) P1-2; (**c**) P1-3; (**d**) P1-4; (**e**) P2-1; (**f**) P2-2; (**g**) P2-3; (**h**) P2-4; (**i**) Untreated wood.

Figure 3 illustrates photos of wood samples before and after being treated with the silicone oil emulsions, with the volume shrink rate (S_v_) and weight percent gain (WPG) of each group presented in Table 2. The top three groups exhibiting the best dimensional stability were P2-2, P2-4, and P1-1. The group P2-2 resulted in a minimal S_v_ of 4.23%, maintaining its shape well with only slight wrinkling. This formulation demonstrated moderate weight gain on the wood, with a WPG of 218.42%. The group P2-4 showed comparable dimensional stability to P2-2, with a S_v_ of 4.93% after dehydration. However, its WPG after treatment was even lower, at 182.37%. The P1-1 also showed an acceptable S_v_ of only 4.60%, but, unfortunately, with significant shape distortion. This formulation also exhibited a low WPG at 180.61%.

The samples in groups P2-2 and P1-2 appeared to exhibit the most natural texture, just like untreated wood. Polymer accumulation on the wood surface was barely visible even on cross-sections and concave areas. In contrast, varying degrees of polymer accumulation were observed in the remaining groups. The excessive polymers mainly caused uneven whitening on the wood surfaces, while the P1-1 group exhibited an unnatural resin-like texture due to the polymer filling inside the cell lumen.

**Figure 4:**
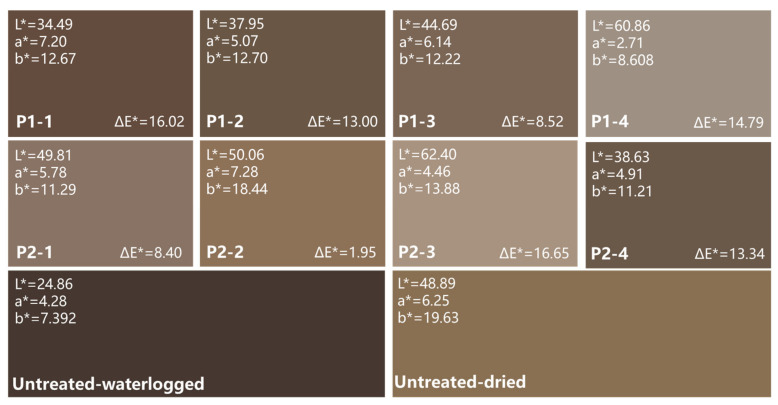
Simulated color according to the CIELab color model of the wood samples.

The simulated color according to the CIELab color model of each group is depicted in Figure 4. All of the dried wood showed a lighter shade compared to the color of waterlogged wood. Compared to untreated air-dried wood, P1-1, P1-2, and P2-4 colors significantly darkened, while P1-4 and P2-3 exhibited pronounced whitening due to a layer of excessive polymers on the surface. The color of P2-2 closely matched that of the untreated air-dried wood, presenting a natural pine wood hue. The colors of the remaining two groups notably shifted towards grey tones.

### 2.3. Vapor Adsorption–Desorption Test

**Figure 5:**
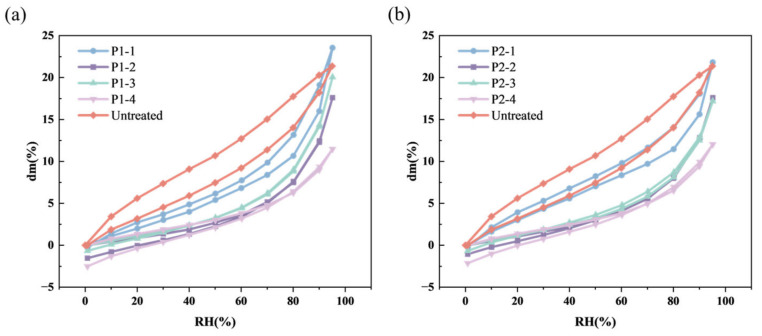
The vapor adsorption–desorption curves at 0% to 95% RH of the wood samples treated with (**a**) P1 and (**b**) P2.

The vapor adsorption–desorption curves at 0% to 95% RH of the wood samples are shown in Figure 5. The untreated wood samples did not exhibit a distinct moisture adsorption–desorption critical point, with a moisture absorption rate of 21% at 95% RH, displaying desorption hysteresis. After desorption, the untreated samples returned to their original weight, indicating reversible moisture adsorption.

We found that the hygroscopicity of the dehydrated wood primarily depended on the surfactants used in the emulsion, but not on the type of silicone oil. Compared to untreated wood samples, the wood treated with the emulsion using STAC (P1-4 and P2-4) had noticeably reduced hygroscopicity, with a maximum moisture absorption rate of 10%. Group P1-1 and P2-1 showed moisture absorption rates at around 21–22.5% at 95% RH, slightly higher than untreated samples. The other two surfactants had no significant impact on the hygroscopicity of the wood, with moisture absorption rates slightly lower than untreated samples at around 17–20% at 95% RH, indicating better environmental stability.

It is also noteworthy that only the groups P1-1 and P2-1 still retained the desorption hysteresis, while all other groups were different. Same as the untreated wood, these treated wood samples were also able to desorb immediately with decreasing humidity, but differed in that their mass was slightly lower than the initial value rather than the same when RH dropped back to 0%. This mass loss of the treated wood was less than 2.5% in all groups.

### 2.4. Microstructural Analysis

**Figure 6:**
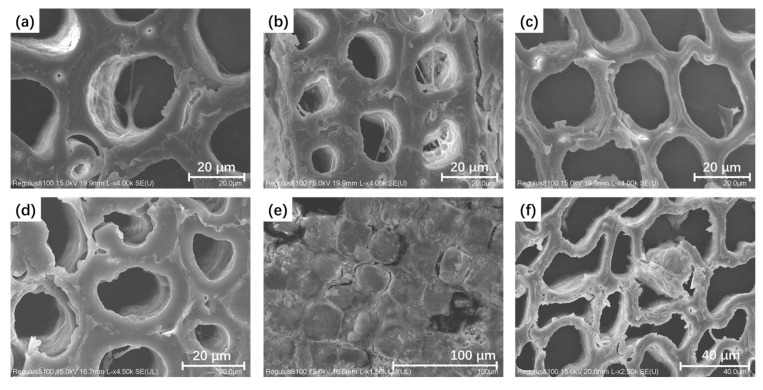
Typical distribution of organic silicon polymerization products in archaeological wood cells (**a**,**b**). Pattern 1: organosilicon polymers formed in the cell lumens in P1-2 and P2-2; (**c**,**d**) pattern 2: organosilicon polymers only adhere to the cell walls of earlywood and latewood cells in P2-2 samples; (**e**) pattern 3: wood cells in P2-1 samples filled with organosilicon polymers; (**f**) the control group samples without silicone oil treatment showed severe deformation of wood cells after being dried in normal conditions.

For the microstructural analysis of wood samples, scanning electron microscopy (SEM) was utilized. The result showed that the silicone oil emulsion, after polymerizing in the wood matrix, displayed a diverse range of microstructural characteristics. This variation led us to identify three typical patterns of polymerized silicone within the wood cells, depicted in Figure 6.

In the majority of the groups, the silicone oil emulsions predominantly entered the wood cell wall and spared the cell lumens from significant filling. Their wood cell walls became denser, which enhanced their strain resistance. Notably, both latewood and earlywood cells maintained their original shapes well with minimal distortion or collapse. Dendritic polymers were found in only a very small percentage of cells, as shown in Figure 6a,b. However, in three groups (P1-3, P1-4, and P2-1), we found the polymers accumulated and filled the cell lumens (Figure 6e).

### 2.5. Chemical Structure

**Figure 7:**
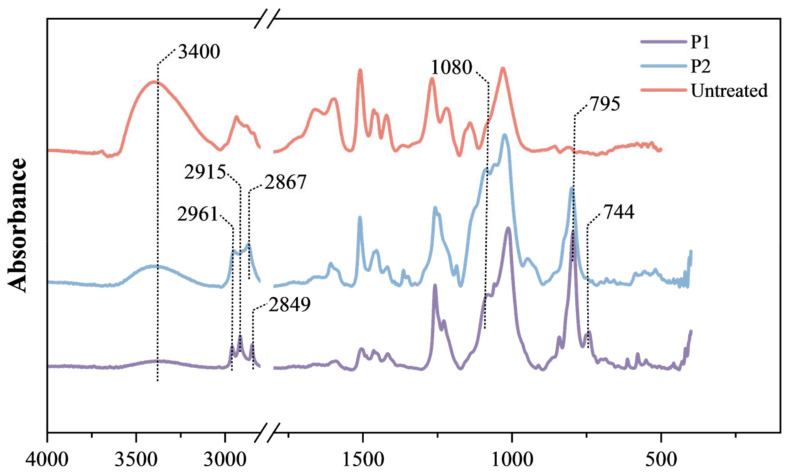
FT-IR spectrum of wood before and after silicone oil emulsions’ dehydration treatment.

To investigate the hydrolysis and condensation reactions within processed wood samples, infrared spectroscopy analysis was conducted. Figure 7 enumerates the primary characteristic peak observed in the infrared spectra pertinent to organosilicon compounds.

Notably, characteristic absorption peaks of Si–O–Si were detected across all sample groups, including antisymmetric and symmetric stretching vibrations at 1080 cm^−1^ and 795 cm^−1^. These peaks confirm the extensive formation of Si–O–Si networks within the wood matrix post hydrolysis and condensation reactions. An additional Si–C stretching vibration absorption peak was observed at 744 cm^−1^. The antisymmetric and symmetric stretching vibration absorption peaks of CH_2_ within long chains were identified at 2915 cm^−1^ and 2849 cm^−1^, respectively, while the terminal CH_3_ stretching vibrations appeared at 2961 cm^−1^ and 2867 cm^−1^. These peaks became pronounced in the spectra of the treated wood samples. Furthermore, a notable decrease in the O–H absorption peak intensity at 3400 cm^−1^.

## 3. Discussion

By comparing the effects of eight groups of silicone oil emulsions on the dimensional stability, moisture resistance, and color of waterlogged archaeological wood samples, this study preliminarily verified the effectiveness of silicone emulsions in the dehydration of waterlogged wooden artifacts. We found that samples with higher WPG generally display lower volume shrinkage, although the S_v_ and WPG did not exhibit an absolute negative correlation. This observation suggested that an adequate material loading capacity may contribute to enhancing the dimensional stability effect. However, the impact of color and appearance also needs to be carefully considered. Overall, considering both physical properties and appearance, P2-2 exhibited the best dehydration effect.

To achieve higher dehydration effectiveness, the selection of surfactants was particularly crucial. In this study, the compatibility of APEO and HPDMS was the best (P2-2), while PDMS did better with APTES or APEO (P1-1 and P1-2). The applicable surfactant can disperse silicone oil droplets more evenly and reduce the viscosity of emulsion, thus making it penetrate wood more efficiently. However, the high-viscosity emulsions using AEO (P1-3 and P2-3) might not play an optimal structural support role due to uneven penetration in wood cells. In addition, we found that using STAC as a surfactant carried a risk of producing white polymers on the surface of the wood, which has a negative impact on the color of the wood after drying (P1-4 and P2-4).

We also tried to reveal the mechanism of silicone oil emulsion changing wood stability from the perspective of chemical structure and microstructure. The SEM analysis results showed that the distribution of organosilicon polymers in wood cells can be divided into three patterns: (i) the polymer appears as a branched network, with a small amount present in the cell lumens; (ii) the polymer only appeared on the cell wall; (iii) the polymer completely filled the cell lumens. An interesting observation was that all groups showing the third pattern exhibited significant shrinkage and polymer enrichment on the wood surface. In contrast, samples presenting patterns 1 and 2 generally maintain their shapes well, such as P2-2 and P1-2. This suggested that excessive silicone polymer filling did not equate to better stabilization. Premature polymerization of silicone oil before its entry into the wood substrate might lead to an uneven distribution of the resulting polymers, affecting its ability to stabilize the cellular structures. The volumetric shrinkage of silicone polymer caused by its excessive polymerization might also paradoxically increase the shrinkage. According to our experimental results, the polymer generated on the cell wall was sufficient to support the cell wall, avoid collapse and deformation during dehydration, and provide a certain degree of moisture resistance. Moreover, it is advantageous to preserve the cell lumen as much as possible for future conservation and restoration works, facilitating the infiltration of reprocessed materials without the need to remove the original dehydration agent.

Absorption peaks of silicone oil polymers were found in the infrared spectra of all samples treated with silicone oil emulsions. Variations in the peak shapes and relative intensities of these absorption features indicate the influence of functional groups from organosilicon molecular chains on the chemical structure of the wood cell walls. Simultaneously, a decrease in the intensity of the hydroxyl absorption peak was observed, which suggested potential chemical cross-linking between the organosilicon compounds and the wood cell walls, leading to a significant reduction in hydroxyl content and absorption water. This finding highlights the transformative impact of silicone oil treatment on the wood cell wall chemical composition. In DVS testing, the disappearance of the desorption hysteresis phenomenon also implied that the generated polymer effectively sealed off the hygroscopic groups on the cell walls and prevented their binding to water, thus improving the stability of the wood artifacts under changing humidity. The mass loss that occurred after desorption was presumably attributed to the incomplete hydrolysis polycondensation reaction of the silicone oil, suggesting that the encapsulation of the silicone by the surfactant limited the degree of polymerization of the silicone oil.

The low cost and the easy availability of silicone oil emulsions afford this dehydration method potential application. However, before applying it to large-sized wooden artifacts in practice, specific application techniques need to be considered in future research. The durability and microbial resistance of reinforced wood require rigorous evaluation. It is also necessary to involve archaeological wood of different tree species and decay degrees.

## 4. Materials and Methods

### 4.1. Archaeological Wood

Archaeological pine wood (*Pinus* sp.) was sourced from the 800-year-old *Nanhai No. 1* shipwreck in the Guangdong Maritime Silk Road Museum (Yangjiang, Guangdong, China) and has been immersed in pure water for four years. The long-time degradation has led to a heavy softening of the wood. The average maximum moisture content (MWC) was 488% and the average basis density was 0.19 g/cm^3^, indicating a severely degraded state distinguished by the international standard [7,35]. Before dehydration, these wood samples were cut into 1 cm^3^ segments and cleaned in pure water.

### 4.2. Emulsion Preparation

This study utilized polydimethylsiloxane (PDMS) and hydroxy-terminated polydimethylsiloxane (PDMS–OH) as the base of the emulsion agents. Four kinds of surfactants were selected, including (3–aminopropyl) triethoxysilane (APTES), alkyl polyoxyethylene ether (APEO), trimethylstearylammonium chloride (STAC), and fatty alcohol polyoxyethylene ether (AEO). A 5 wt% of 2–(2–butoxyethoxy) ethanol was used as a cosurfactant, and a 0.5 wt% of dibutyltin dilaurate (DBTL) was introduced as the catalyst. Comprehensive details on the application of these chemical reactants are outlined in Table 3.

Emulsion agents were formulated with 30 wt% of silicon oil, 10–15 wt% of surfactants, and 5 wt% of cosurfactant. The formulations are exhibited in Table 4. The preparation steps of the emulsion were as follows (shown in Figure 7): (i) mix surfactants and pure water uniformly into the solution A; (ii) combine the silicone oil with the cosurfactant to form the solution B; (iii) gradually drip the solution B into A, while stirring at 5000 rpm for 20 min; (iv) increase the stirring speed to 8000 rpm for 5 min, until the formation of a clear or milky white water-in-oil emulsion. The resultant emulsion should readily dissolve in water and remain stable upon standing for at least 24 h. The viscosity of the emulsion is tested to prevent excessive pre-polymerization during preparation.

### 4.3. Dehydration of Wood

Each group of wood samples of 1 cm^3^ was separately immersed in the prepared silicone oil emulsions for three days. Subsequently, a 0.5 wt% DBTL was introduced into the emulsion, and the immersing process was sustained for an additional two days. After that, gels on the wood surface were cleaned in water, and samples were then left to dry under normal conditions (at 25 °C with a relative humidity of 34.5 ± 1.5%) until the weight remained constant.

### 4.4. Testing and Characterization Methods

Photos of the wood samples were taken at 20× magnification to compare their overall appearance before and after treatment using a three-dimensional video microscope (VHX-6000, KEYENCE, Osaka, Japan).

The Basic Density (BD) and Maximum Water Content (MWC) of wood were calculated according to Equation (1) and Equation (2), respectively. The mass of the waterlogged sample with the size of about 10 mm *×* 10 mm *×* 10 mm was measured with an analytical balance (JA2003, SUNNY HENGPING Instrument, Shanghai, China) and recorded as $M_{1}$. After being dried in an oven at 103 *±* 2 °C, the mass of the sample was recorded as $M_{0}$. The drainage method was used to measure the waterlogged volume $V_{1}$ and dry volume $V_{0}$ under normal conditions. On the analytical balance, the sample was completely immersed in pure water without touching the bottom and wall of the beaker. According to Archimedes’ principle, the volume of the wood sample (cm^3^) equals the increased weight (g) in value of the pure water.

The weight percent gain (WPG) was calculated using Equation (3), and the anti-shrinkage effect of the treatment was evaluated using the volume shrink rate (S_v_) calculated with Equation (4). In Equation (3), *m*_1_ is the dry mass of the wood after treatment and reaching equilibrium in a normal environment, and *m*_0_ is the theoretical dry mass of the wood sample, which is estimated according to the $M_{1}$ and MWC.

(1) $$BD=\frac{M_{0}}{V_{1}}\;\;$$

(2) $$MWC=\frac{M_{1}-M_{0}}{M_{0}}\;$$

(3) $$WPG=\left(m_{1}-m_{O}\right)\times 100\%$$

(4) $$S_{V}=\frac{V_{1}-V_{0}}{V_{1}}\times 100\%$$

The color difference (ΔE*) between the dried samples before and after treatment with the emulsions was measured with a spectrophotometer (CM-26D, Konica Minolta, Tokyo, Japan) under a D65 light source. The following equation was used to calculate the color difference ΔE*:

ΔE* = [(ΔL*)^2^ + (Δa*)^2^ + (Δb*)^2^]½, (5)

where L* ranges from 0 to 100 and indicates the change in color from black (dark) to white (light), a* varies from negative to positive and represents the change in color from green to red, and b* ranges from negative to positive and indicates the change in color from blue to yellow.

In the scanning electron microscope (SEM) analysis, an ultra-high-resolution field-emission scanning electron microscope (Regulus 8100, HITACHI, Tokyo, Japan) was used. Gold was sputtered to the surfaces of samples as a conductive coating. The microscope was operated with an accelerating voltage of 15 kV, a resolution of 0.7 nm, a working distance of 15 mm, and a secondary electron imaging (SE) working mode.

The maximal moisture content under different relative humidity (RH) conditions was measured using a high-throughput dynamic moisture-adsorption tester (SPSx-1μ, ProUmid, Ulm, Germany). Preceding the test, the samples were air-dried at 103 °C for more than 24 h until their weights no longer decreased. The humidity scope of this test ranged from 0 to 95% RH, at a gradient of 10% RH and a stable temperature of 25 °C. The default measurement frequency was every 10 min. The time taken for every gradient fell between a minimum of 60 min and a maximum of 600 min. The default weight limit had an upper cap of 1000%. The equilibrium condition was defined as dm/dt ≤ 0.01.

In chemical structure analysis, wood samples were tested using a Fourier transform infrared spectrometer (Nicolet™ iS™5, Thermo Scientific, Waltham, MA, USA) with 32 scanning times and a resolution of 4.000 cm^−1^. The absolute dry mass ratio of the wood sample to potassium bromide powder was 1:100.

## 5. Conclusions

The biggest challenge in achieving controllable and in situ polymerization of organosilicon is how to regulate the contradiction between organosilicon and water. Water-repellent organosilicon materials cannot enter the interior of the wood through the waterlogged pores and will initiate polymerization reactions once they encounter moisture, which makes the dehydration and shaping process uncontrollable.

In this study, low-molecular-weight silicone oil was prepared into oil in water emulsions, which realized the direct replacement of water in wood without organic solvents. After entering the wood cells, silicone oil tended to form on the cell wall and undergo slow polymerization. The generated organic silicon polymer effectively supported and strengthened the wood cell wall during the dehydration process.

Among the eight groups of silicone oil emulsions, P2-2 showed the best efficiency, with a shrinkage rate of only 1.63%, and the appearance of the wood is also very natural. The weight gain rate was only 218%, which was conducive to reducing the burden of external consolidants on the wooden artifacts. Overall, the filling degree of the cell lumen via organosilicon polymers was limited. Under SEM, it can be seen that the silicone oil only polymerized on the wood cell wall, with almost no filling in the cell lumen. The wood cell wall was well shaped, without severe deformation or collapse like the untreated sample.

This study indicated that this in situ polymerization reinforcement method has a good stabilizing effect on the shape of waterlogged archaeological wood. In addition, it is significant for the restoration of cultural relics that this method can effectively preserve the pore structures of wood cells so that retreatment materials can still penetrate the wooden artifacts in the future.

## Figures and Tables

**Table 1:** Viscosity of the silicone oil emulsions.

Group	P1-1	P1-2	P1-3	P1-4	P2-1	P2-2	P2-3	P2-4
Viscosity/mPa·s	54.0	76.5	105.0	21.5	49.5	68.0	89.5	26.5

**Table 2:** The weight percent gain (WPG) and volume shrink rate (Sv) of pine wood samples treated with silicone oil emulsions.

Group	Sv	WPG
P1-1	4.60%	180.61%
P1-2	N/A *	213.16%
P1-3	14.09%	126.93%
P1-4	13.34%	110.74%
P2-1	16.16%	179.49%
P2-2	4.23%	218.42%
P2-3	N/A *	155.59%
P2-4	4.93%	182.37%

* Accurate data were not obtained due to the destruction of severely cracked samples.

**Table 3:** Materials used for the silicone oil emulsion.

Materials	Mw (g/mol)	Molecular Formula	Manufacturer/Purity
Polydimethylsiloxane (PDMS)	~770	[–Si (CH_3_)_2_O–]_n_	Sigma-Aldrich/99%
Hydroxy terminated polydimethylsiloxane (PDMS-OH)	400~700	HO–(C_2_H_6_OSi)_n_–H	Gelest/99%
2–(2–Butoxyethoxy) ethanol	162.23	C_8_H_18_O_3_	Aladdin/99%
Dibutyltin dilaurate (DBTL)	631.56	C_32_H_64_O_4_Sn	Aladdin/95%
(3–Aminopropyl)triethoxysilane (APTES)	221.37	C_9_H_23_NO_3_Si	Aladdin/99%
Alkyl polyoxyethylene ether (APEO)	N/A	HO(CH_2_CH_2_O) _n_(CH_2_)_11_CH_3_	Aladdin/99.5%
Trimethylstearylammonium chloride (STAC)	348.05	C_21_H_46_ClN	Aladdin/98%
Fatty alcohol polyoxyethylene ether (AEO)	315/590	RO–(CH2CH2O) _n_–H	Aladdin/98%

**Table 4:** Formulations of the silicone oil emulsion.

Group	Silicone Oil	Surfactant
P1-1	30% PDMS	10% APTES
P1-2		15% APEO
P1-3		15% AEO
P1-4		10% STAC
P2-1	30% PDMS-OH	10% APTES
P2-2		15% APEO
P2-3		15% AEO
P2-4		10% STAC

## Data Availability

The data presented in this study are available on request from the corresponding author.
